# CRISPR-Cas3 and type I restriction-modification team up against *bla*_KPC_-IncF plasmid transfer in *Klebsiella pneumoniae*

**DOI:** 10.1186/s12866-024-03381-7

**Published:** 2024-07-03

**Authors:** Yang Yang, Peiyao Zhou, Dongxing Tian, Weiwen Wang, Ying Zhou, Xiaofei Jiang

**Affiliations:** 1 Institute of Antibiotics, Huashan Hospital, Fudan University, Shanghai, China; 2 Key Laboratory of Clinical Pharmacology of Antibiotics, Ministry of Health, Shanghai, China; 3 Department of Laboratory Medicine, The First Affiliated Hospital of Wenzhou Medical University, Wenzhou, China; 4 Department of Laboratory Medicine, Shanghai Medical College, Huashan Hospital, Fudan University, Shanghai, China; 5 Department of Clinical Laboratory Medicine, Shanghai Pulmonary Hospital, Tongji University School of Medicine, Shanghai, People’s Republic of China

**Keywords:** CRISPR-Cas system, Type I R-M systems, *bla*_KPC_ harbouring plasmids, *Klebsiella pneumoniae* carbapenemase (KPC)-producing *Klebsiella Pneumoniae*, Horizontal gene transfer

## Abstract

**Objective:**

We explored whether the Clustered regularly interspaced short palindromic repeat (CRISPR)-Cas and restriction-modification (R-M) systems are compatible and act together to resist plasmid attacks.

**Methods:**

932 global whole-genome sequences from GenBank, and 459 *K. pneumoniae* isolates from six provinces of China, were collected to investigate the co-distribution of CRISPR-Cas, R-M systems, and *bla*_KPC_ plasmid. Conjugation and transformation assays were applied to explore the anti-plasmid function of CRISPR and R-M systems.

**Results:**

We found a significant inverse correlation between the presence of CRISPR and R-M systems and *bla*_KPC_ plasmids in *K. pneumoniae*, especially when both systems cohabited in one host. The multiple matched recognition sequences of both systems in *bla*_KPC_-IncF plasmids (97%) revealed that they were good targets for both systems. Furthermore, the results of conjugation assay demonstrated that CRISPR-Cas and R-M systems in *K. pneumoniae* could effectively hinder *bla*_KPC_ plasmid invasion. Notably, CRISPR-Cas and R-M worked together to confer a 4-log reduction in the acquisition of *bla*_KPC_ plasmid in conjugative events, exhibiting robust synergistic anti-plasmid immunity.

**Conclusions:**

Our results indicate the synergistic role of CRISPR and R-M in regulating horizontal gene transfer in *K. pneumoniae* and rationalize the development of antimicrobial strategies that capitalize on the immunocompromised status of KPC-KP.

**Supplementary Information:**

The online version contains supplementary material available at 10.1186/s12866-024-03381-7.

## Introduction

*Klebsiella pneumoniae* carbapenemase (KPC)-producing *K. pneumoniae* (KPC-KP) has emerged as an urgent threat to global public health because of its prevalence and associated high mortality rate. Therefore, controlling the dissemination of KPC-KP has become a critical global issue [1]. Of epidemiological significance, the international pandemic of KPC-KP is primarily associated with *bla*_KPC_ harbouring plasmids, with *bla*_KPC_-IncF plasmids (IncF *bla*_KPC_-harbouring plasmids) being dominant [1, 2].

In our previous work, we proposed a mechanism for the emergence of the KPC-KP. We found that these strains lack endogenous barriers to horizontal gene transfer (HGT), including clustered regularly interspaced short palindromic repeat (CRISPR)-Cas systems and restriction-modification (R-M) systems [3–5]. Antibiotic use inadvertently selects for the outgrowth of these immunocompromised strains with enhanced abilities to acquire resistant elements (such as *bla*_KPC_ plasmid), thereby assisting their rapid adaptation to antibiotic-treated patients and the hospital environment [3–5].

By analysing the whole genome sequences of a series of *K. pneumoniae*, only type I-E CRISPR systems in chromosomes were identified (dataset) [3, 4, 6]. We previously revealed a significant inverse correlation between the presence of type I-E CRISPR systems and *bla*_KPC_ harbouring plasmids in clinical *K. pneumoniae* isolates [3, 4]. The mechanism of type I-E CRISPR-Cas genome defense has been recently reviewed. The system encodes the CRISPR-associated complex for antiviral defense (cascade) complex to bind to a *bona fide* target (proto-spacer) upon PAM (proto-spacer adjacent motif) recognition and subsequently recruits the Cas3 protein to execute DNA cleavage [7–9](Figure S1).

Restriction-modification (R-M) systems provide another form of genome defense by acting as barriers to HGT through self-recognition versus non-self-recognition of methylation signatures [10, 11]. By analysing 932 whole genome sequences of *K. pneumoniae* in GenBank, we focused on the type I R-M systems (dataset) and observed a remarkable inverse correlation between the presence of type I R-M systems and that of *bla*_KPC_ harbouring plasmids [5]. A type I restriction enzyme comprises three subunits encoded by three closely linked genes: *hsdR* (HsdR, restriction), *hsdM* (HsdM, methyltransferase), and *hsdS* (HsdS, conferring target sequence specificity) [5, 10]. Type I R-M systems are also known for their diversity and the relative ubiquity of their recognition sequences in prokaryotes [10]. We have previously classified type I R-M systems of *K. pneumoniae* into one of five discrete families: type IA, IB, IC, ID, and Im6A. Among these R-M families, those in type IC were plasmid-borne, and those belonging to types IA, IB, and ID were specified in the bacterial chromosome. The linkage between IC R-M units and *bla*_KPC_-plasmids suggested that these systems would treat related resistant genes as self-DNA elements, not attacking (restriction) but protecting (methylation); hence, we categorized IC R-M systems of *K. pneumoniae* as immunocompromised [5]. Notably, others lack HsdR (HsdMS, Im6A), which has only methyltransferase activity but offers no barrier to *bla*_KPC_ invasion [5].

Despite being two of the most well-studied defense systems, which often cohabit in the same host, only a few studies have explored the possibility of interactions between the R-M and CRISPR-Cas systems [12, 13]. Although we have demonstrated that CRISPR-Cas and R-M systems have the potential to limit the entry of *bla*_KPC_ genes [3–5], epidemiological and functional data supporting the synergistic role of both systems in rapidly spreading high-risk KPC-KP remain insufficient. Moreover, a bacterium encodes more than one defense system, raising the possibility of interactions between defense mechanisms in plasmid infection. We aimed to explore these potential interactions to unveil the existence of complex immune strategies in bacteria.

In this study, we collected 932 global whole-genome sequences from GenBank, and 459 *K. pneumoniae* isolates from six provinces of China, to investigate the co-distribution of CRISPR-Cas and type I R-M systems. We also studied the distribution of both defense systems and *bla*_KPC_ genes, to clarify the relationship between the team-up activity of such systems and *bla*_KPC_ dissemination in *K. pneumoniae.* We also analyzed whether the dominant *bla*_KPC_ harbouring plasmids (*bla*_KPC_-IncF plasmids) would be good targets for both the CRISPR and R-M systems. Finally, we used the conjugation assay to deeply explore the anti-plasmid function of CRISPR and R-M alone and the potential coupling function of both systems in perturbing the dissemination of the *bla*_KPC_ harbouring plasmids. This study is the first step toward understanding the interaction between CRISPR and R-M systems in regulating HGT in *K. pneumoniae*.

## Materials and methods

### *K. pneumoniae* isolates

All global *K. pneumoniae* complete genome sequences in this study (932 in total) are publicly available and downloaded from the NCBI database in May 2022 (dataset, sheet1). The plasmid incompatibility type was identified by comparison with the information in the plasmid MLST locus/sequence definitions database (https://pubmlst.org/bigsdb?db=pubmlst_plasmid_seqdef). The acquired antibiotic resistance genes were identified using ResFinder (https://cge.cbs.dtu.dk/services/ResFinder/), with the default threshold. Kleborate v2.0 (https://github.com/klebgenomics/Kleborate), was used to screen genome assemblies for MLST, capsular type, and virulence loci. Moreover, we also randomly collected 459 non-duplicated *K. pneumoniae* isolates from individual patients at seven hospitals in six provinces of China. The presence of *bla*_KPC_ in these strains was determined using the pair of primers listed in Table S1. Multilocus sequence typing (MLST) was performed according to the protocol described on the Pasteur Institute MLST website for *K. pneumoniae*.

### Prevalence of type I-E CRISPR-Cas and type I R-M systems

For the 932 global *K. pneumoniae* isolates extracted from the NCBI database, CRISPR-Cas finder [14](https://crisprcas.i2bc.paris-saclay.fr/CrisprCasFinder/Index) was used with default parameters to identify the Cas operon and CRISPR loci in the genomes (dataset, sheet1 and sheet3) and determine the number and sequences of spacers within the CRISPR repeat arrays. The components of R-M systems were obtained from the REBASE database (http://rebase.neb.com) [15]. For the 459 clinical *K. pneumoniae* isolates, the prevalence of CRISPR-Cas and R-M was determined using polymerase chain reaction with primers described previously (Table S1) [4, 5]. Nucleotide BLAST was also used to search for matched proto-spacers and R-M recognition sites with a minimum of 90% homology to *bla*_KPC_-IncF plasmids (dataset). The IncFII_K_ plasmid, p187-2 (GenBank accession No. CP025468.1) from our previous studies, was used as a reference plasmid for comparison. CGView (http://cgview.ca/) was used to visualize the IncF plasmids to exemplify the conservative backbone sequences targeted by the CRISPR-Cas and R-M systems (E-value, *≤* 0.1).

### Generation of isolates harbouring CRISPR-Cas or R-M used in this study

The pACYC-KP8CRISPR was assembled with three fragments: the two fragments (with 50-bp overlap) of the CRISPR-Cas system were amplified from KP8 ((type I-E, CP025636.1), being *bla*_KPC_-negative and belonging to ST458, was used as a model owing to its abundant matched spacers), and the Pi-dependent plasmid backbone (Kana^R^) was amplified from pACYC-184 using primers with 30-bp homology to the CRISPR-Cas fragment of KP8 (Table S1). Plasmid fragments were combined using the in-fusion cloning method and NEBuilder HiFi DNA assembly master mix (New England BioLabs). The pACYC-KP8CRISPR plasmid was then transformed into *E. coli* NM1049 (Type IA RM system, *EcoKI*, (AACNNNNNNGTGC), CRISPR-, Kana^R^), NK354(Type IB RM system, *EcoAI* (GAGNNNNNNNGTCA), CRISPR-, Kana^R^), NM10091(Type ID RM system, *StySBLI* (GGTANNNNNNTCG), CRISPR-, Kana^R^), and NM1261(RM system null, HsdS-, CRISPR-, Kana^R^) [16] to produce an effective CRISPR system and resistance to kanamycin. Moreover, as all isolates harbouring type I R-M systems, including the null control, lacked a suitable resistance marker, the plasmid pACYCY-184-Kana^R^ was transformed to them, thereby conferring resistance to kanamycin. All isolates are listed in Table 1.

**Table 1:** Strains information

Strain	Characteristics	Reference
Donor		
JS531	*E. coli* Top10 harboring p187-2 plasmid (an IncF conjugative plasmid isolated from *K. pneumoniae*, Amp^R^, GenBank Accession No. CP025468.1)	[17]
Recipients		
NM1049	*E. coli* DE3, Type IA RM system, *EcoKI*, (AACNNNNNNGTGC), CRISPR-, Kana^R^	[16]
NK354	*E. coli* DE3, Type IB RM system, *EcoAI* (GAGNNNNNNNGTCA), CRISPR-, Kana^R^	[16]
NM1009	*E. coli* DE3, Type ID RM system, *StySBLI* (GGTANNNNNNTCG), CRISPR-, Kana^R^	[16]
NM1261	RM system null, HsdS-, CRISPR-, Kana^R^	[16]
JS696	*E.coli*, NM1261 harboring plasmid pACYC-KP8CRISPR, RM-, KP8-CRISPR+, Kana^R^	This study
JS697	*E.coli*, NM1049 harboring plasmid pACYC-KP8CRISPR, Type IA RM system+, KP8-CRISPR+, Kana^R^	This study
JS698	*E.coli*, NK354 harboring plasmid pACYC-KP8CRISPR, Type IB RM system+, KP8-CRISPR+, Kana^R^	This study
JS700	*E.coli*, NM1009 harboring plasmid pACYC-KP8CRISPR, Type ID RM system+, KP8-CRISPR+, Kana^R^	This study

### Conjugation experiment

*E. coli* isolates harbouring CRISPR or R-M were constructed as recipient strains (Table 1). Cultures of the donor strain *E. coli* JS531 containing the plasmid p187-2 (Table 2, an IncF conjugative plasmid isolated from *K. pneumoniae*, Amp^R^), which harbours *bla*_KPC−2_ and matches the recognition sites of CRISPR and R-M systems, and recipient (Kana^R^) cells in logarithmic phase (1 mL) were mixed at a ratio of 1:1, and then resuspended in 20 µL MgSO4 (10 mM). The suspension was spotted on a Luria-Bertani (LB) plate and incubated at 37 °C overnight. After 20-hour static incubation at 37 °C, serial dilutions were plated in the appropriate media (100 mg/L ampicillin and 50 mg/L kanamycin). All transconjugants were confirmed by screening for the *bla*_KPC_ gene using the primers listed in Table S1. The conjugation frequency was calculated as conjugants per recipient. All conjugation experiments were performed in triplicate.

**Table 2:** Recognition information of p187-2 plasmid

p187-2	KP8-CRISPR					Type I *R*-M		
	spacer1	spacer3	spacer4	spacer5	spacer6	IA	IB	ID
Numbers	2	1	1	3	1	12	13	11

### Statistics

Statistical significance was assessed by one-way analysis of variance (ANOVA) using GraphPad Prism9 software.

## Results

### The co-distribution of type I-E CRISPR-Cas and type I R-M systems in *Klebsiella pneumoniae*

Our previous studies also confirmed the wide distribution and pleomorphism of type I-E CRISPR-Cas and type I R-M systems in *Klebsiella pneumoniae*, but the ratio of genomes that co-encode both systems has not been clarified. A total of 932 global latest *K. pneumoniae* whole-genome sequences available in GenBank databases (dataset) and 459 *K. pneumoniae* isolates from six provinces of China (dataset) were analyzed to determine the distribution of CRISPR-Cas and type I R-M systems.

**Fig. 1:**
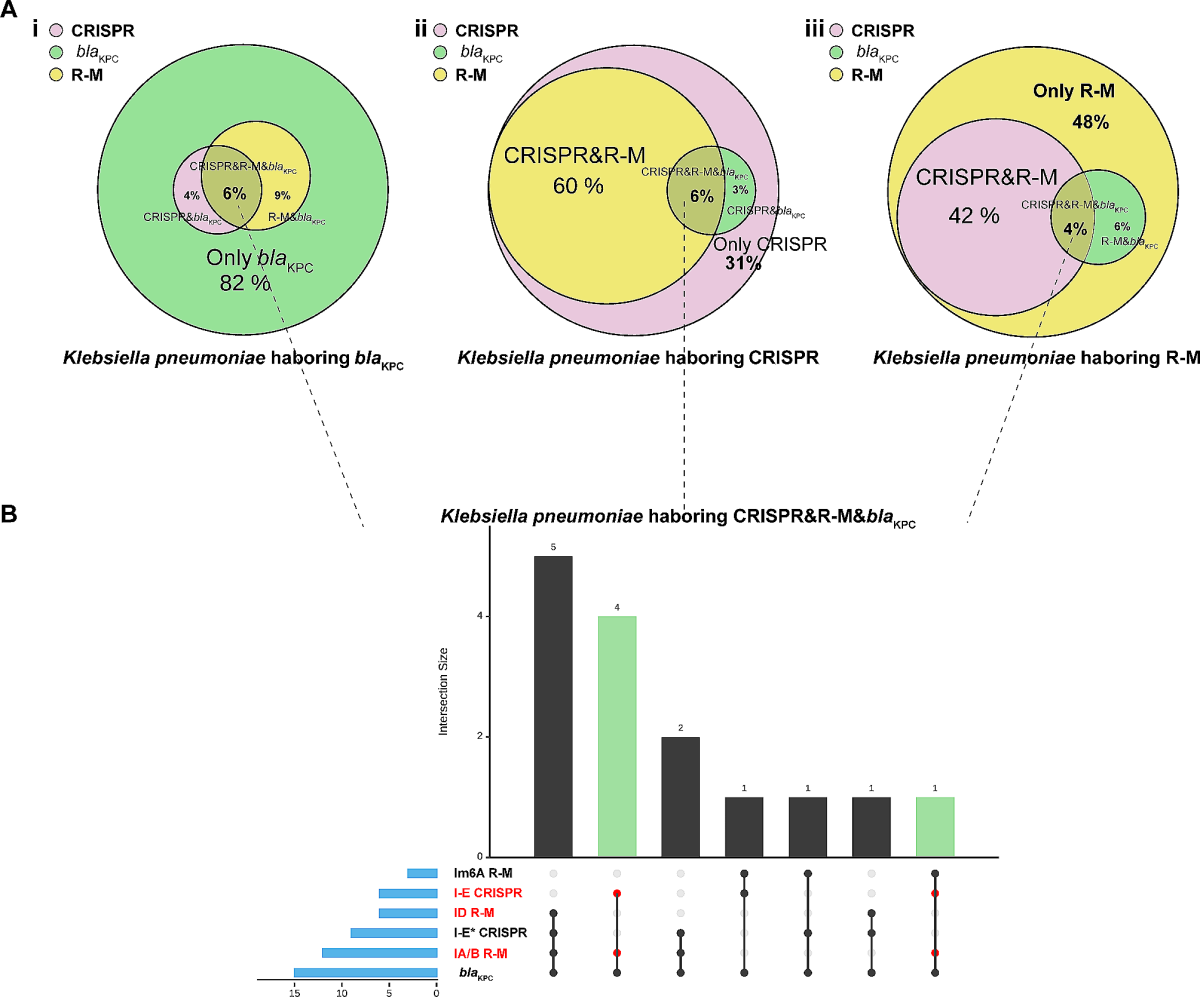
Distribution of CRISPR-Cas systems, R-M systems, and *bla*_KPC_ in 932 completely sequenced global *Klebsiella pneumoniae***(A)** Presence of CRISPR-Cas systems, R-M systems, and *bla*_KPC_ in 271 *bla*_KPC_ harbouring isolates in (i), 247 CRISPR-positive isolates, and 360 R-M positive isolates. **(B)** Distribution of different types of CRISPR-Cas and R-M systems in *Klebsiella pneumoniae* co-harbouring CRISPR, R-M and *bla*_KPC_

We found that the CRISPR-Cas and type I R-M systems were rare (17.4%) in both KPC-KP and carbapenem-resistant *Klebsiella pneumoniae*, but abundant (54.4%) in *bla*_KPC_-negative isolates (Fig. 1Ai and S2, dataset), which was consistent with our previous findings. Moreover, type I R-M systems were more widely present in sequenced *K. pneumoniae* genomes than CRISPR-Cas (360/932 vs. 247/932), indicating that R-M systems may confer more defense to prevent *bla*_KPC_ invasion (dataset). As previously described, one bacterium always harbours multiple anti-plasmid strategies. Among the CRISPR-positive isolates in this study, approximately 60% of the strains held type I R-M systems simultaneously (Fig. 1Aii). For the R-M-positive strains, immune strategies were more complex; we observed that approximately 42% of the isolates possessed CRISPR systems; however, approximately 30% of the strains encoded more than one type I R-M systems (Fig. 1Aiii, dataset). Many genomes encode both systems, raising questions regarding their interactions.

When only one defense strategy was present in the isolates, we still detected some *bla*_KPC_-escapers. However, we only observed that 1–2% (15/932;10/459) of isolates carried both CRISPR and R-M systems, and *bla*_KPC_ genes (Fig. 1B and S1). Notably, the presence of Im6A/IC R-M systems (immunocompromised R-M systems) or I-E* CRISPR-Cas systems (low-matched spacers) were the main factor causing this phenomenon. In other words, the co-existence of such immune systems does not achieve functional interaction between the two systems, and only one effective system plays a defensive role. When *K. pneumoniae* co-carried IA/B/D R-M systems and I-E CRISPR-Cas system, the detection rate of *bla*_KPC_ approached 0 (Fig. 1B and S1). These results indicate that the co-existence of the IA/B/D R-M systems and I-E CRISPR-Cas system may enhance immune function and influence the dissemination of *bla*_KPC_ harbouring plasmids.

### *bla*_KPC_-IncF plasmids can be well-targeted by both CRISPR and R-M systems in *K. pneumoniae*

The relationship between CRISPR and R-M systems and the occurrence of *bla*_KPC_ harbouring plasmids in *K. pneumoniae* have been investigated [3–5]. We evaluated whether *bla*_KPC_-harbouring plasmids were effectively targeted by CRISPR and R-M systems. We analyzed the distinct spacers and recognition sites in 932 globally sequenced *K. pneumoniae* genomes, identified 247 CRISPR loci (dataset), and obtained 18 matched proto-spacers, as previously described [3, 4], in the *bla*_KPC_ harbouring plasmids extracted from these isolates. Moreover, we also identified eight major R-M clades and four main recognition sites (“GAAYNNNNNNNCTGG,” “ACANNNNNNNNTGAC,” “CATCNNNNNNTTYG,” and “ACGNNNNNGTTG”) in IA/B/D R-M systems [5].

**Fig. 2:**
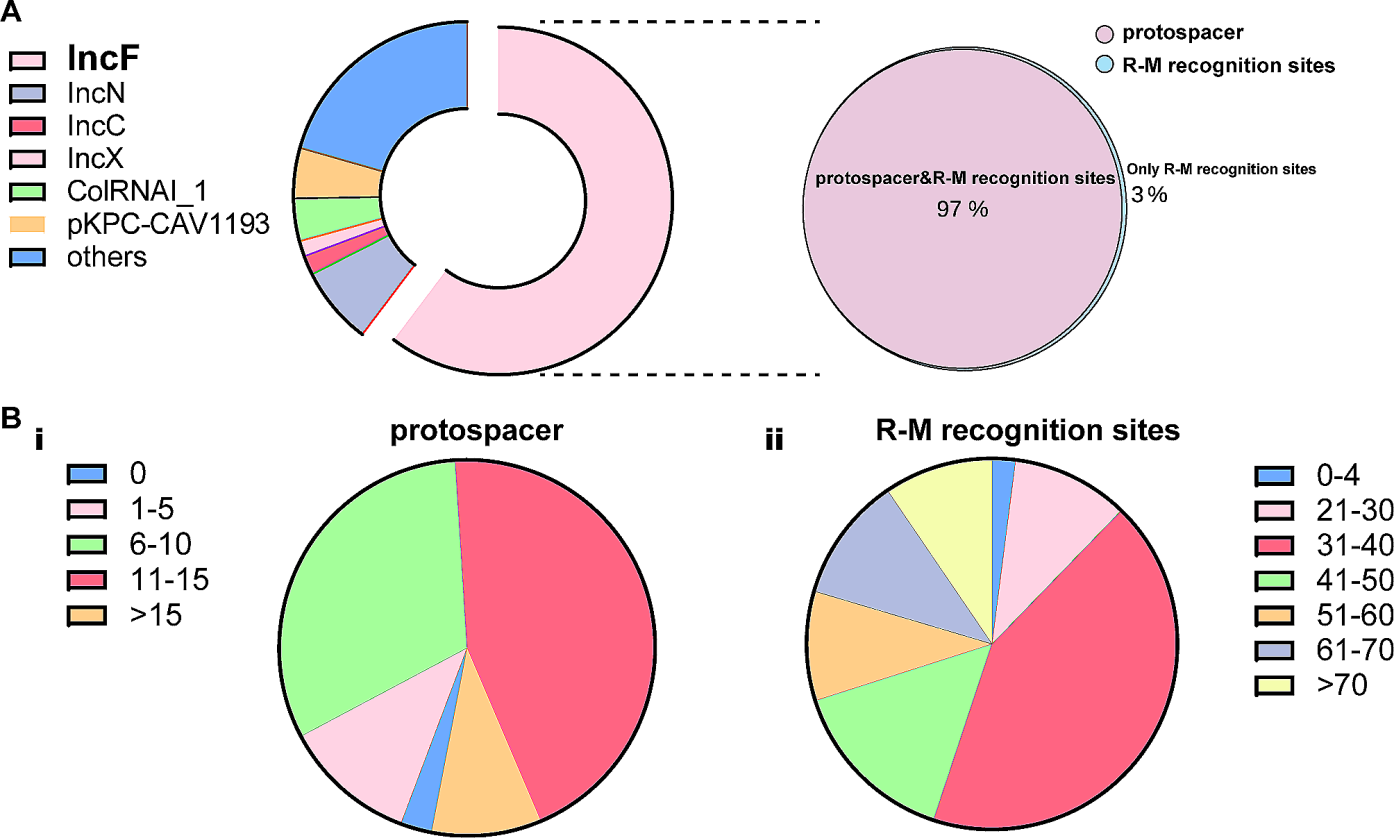
Characteristics of *bla*_KPC_ harbouring plasmids extracted from 932 global *Klebsiella pneumoniae*. **(A)** The incompatibility type distribution of *bla*_KPC_ harbouring plasmids. **(B)** The number of proto-spacers (i) and R-M recognition sites among IncF *bla*_KPC_ harbouring plasmids (ii)

**Fig. 3:**
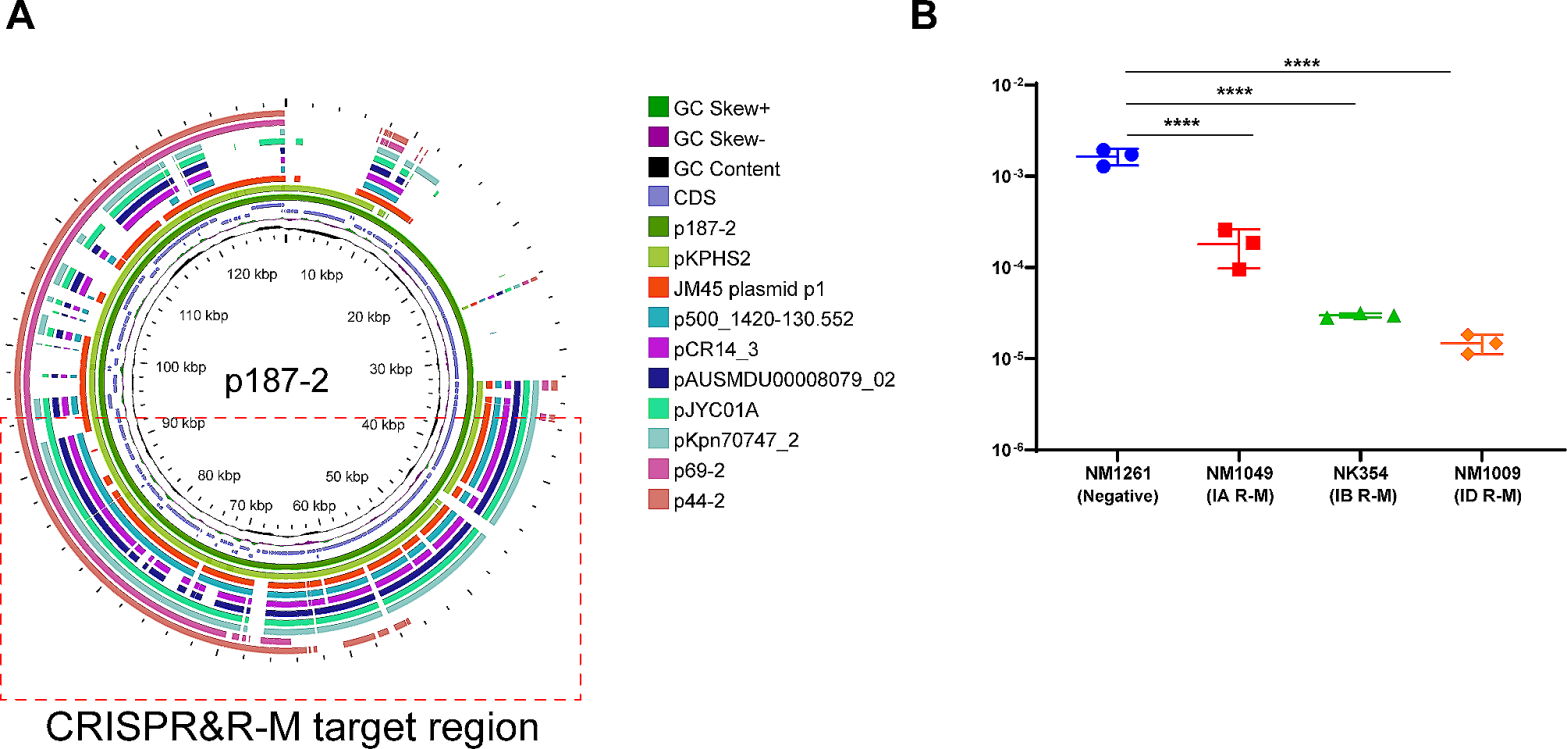
Conjugation frequencies of p187-2 plasmid in strains with or without type I R-M systems. **(A)** Comparative analysis of IncF *bla*_KPC_ harbouring plasmids using p187-2 as the reference. The CRISPR and R-M targeted regions are illustrated with red dotted boxes. The detailed information on these plasmids is listed in the dataset. **(B)** Type I R-M systems influence the conjugation of the p187-2 plasmid. The data represent the mean ± SD for three independent biological replicates. ****p < 0.0001 indicate significant differences between strains and the control group as determined using one-way ANOVA with Dunnett correction

In *K. pneumoniae*, the IncF plasmid is the most predominant plasmid incompatibility type and plays a critical role in the worldwide dissemination of *bla*_KPC_ in *K. pneumoniae* [2, 18]. Our analysis showed that IncF plasmids serve as a dominant vector (~ 60%) for *bla*_KPC_ genes (Fig. 2A). Hence, we searched for matched sequences on all *bla*_KPC_-IncF plasmids to determine whether CRISPR and R-M systems are associated with the dissemination of *bla*_KPC_. Of the *bla*_KPC_-IncF plasmids extracted from 932 global *K. pneumoniae*, 97% of plasmids were identified as being targeted with more than one spacer and R-M recognition sites. Consistent with our previous findings, 85.7% of *bla*_KPC_-IncF plasmids were matched with more than six spacers, and 97.9% of plasmids were targeted by more than 20 R-M recognition sites (Fig. 2B). These target sequences (including spacers and R-M recognitions) were were conservative and related to plasmid carriage and propagation (dataset, Fig. 3A). These findings indicate that the dominant *bla*_KPC_-IncF plasmids are inhibited by both CRISPR and R-M systems in *K. pneumoniae.*

### Type I R-M systems reduce the conjugative transfer of *bla*_KPC_-IncF plasmid

The paucity of the IA/B/D R-M system in *bla*_KPC_-positive *K. pneumoniae* suggests that R-M units may be involved in preventing the acquisition of *bla*_KPC_ harbouring plasmids. Hence, we used conjugation assays to determine whether the type I R-M system was disadvantageous to the dissemination of *bla*_KPC_ plasmids. Type I R-M systems in *Enterobacteriales* are diverse and subdivided into families (such as IA/B/D), which are currently defined by DNA hybridization, subunit complementation, antibody cross-reactivity, and sequence conservation. For each family, the functional domains of HsdM (modification) and HsdR (restriction) are conserved, and the essential difference between the two members of one family resides in the regions of the HsdS subunit that confer sequence specificity [10, 11, 19, 20].

We selected isolates carrying classical and well-studied type IA/B/D R-M systems identified as recipients in *Enterobacteriales* to confirm whether type I R-M systems could restrict *bla*_KPC_ dissemination. Moreover, one isolate lacking R-M units was used as a negative control (NM1261), and JS531 [17] were used as the donor. According to a previous analysis, IncF-type plasmids were the dominant vectors for *bla*_KPC_ gene. Moreover, the targeted sequences of the two defense systems corresponded with the plasmid stability regions and conjugation modules, which were conserved in the IncF plasmids (Dataset sheet 6, Fig. 3A) [18]. Hence, the typical *bla*_KPC_-IncF plasmid p187-2 selected in this study could reflect immune interference for *bla*_KPC_ in the CRISPR and R-M systems.

The conjugation frequencies of the three recipients were compared with those of the negative controls. As shown in Fig. 3B, the R-M systems confer over 1-log reduction in the acquisition of the *bla*_KPC_ plasmid. For the IB and ID families, the frequencies were reduced by 2-log. These results show that type I R-M had a significant impact on conjugative *bla*_KPC_-IncF plasmid transfer in bacteria.

### Type I R-M and CRISPR-Cas systems exhibited no significant difference in hindering *bla*_KPC_ invasion

**Fig. 4:**
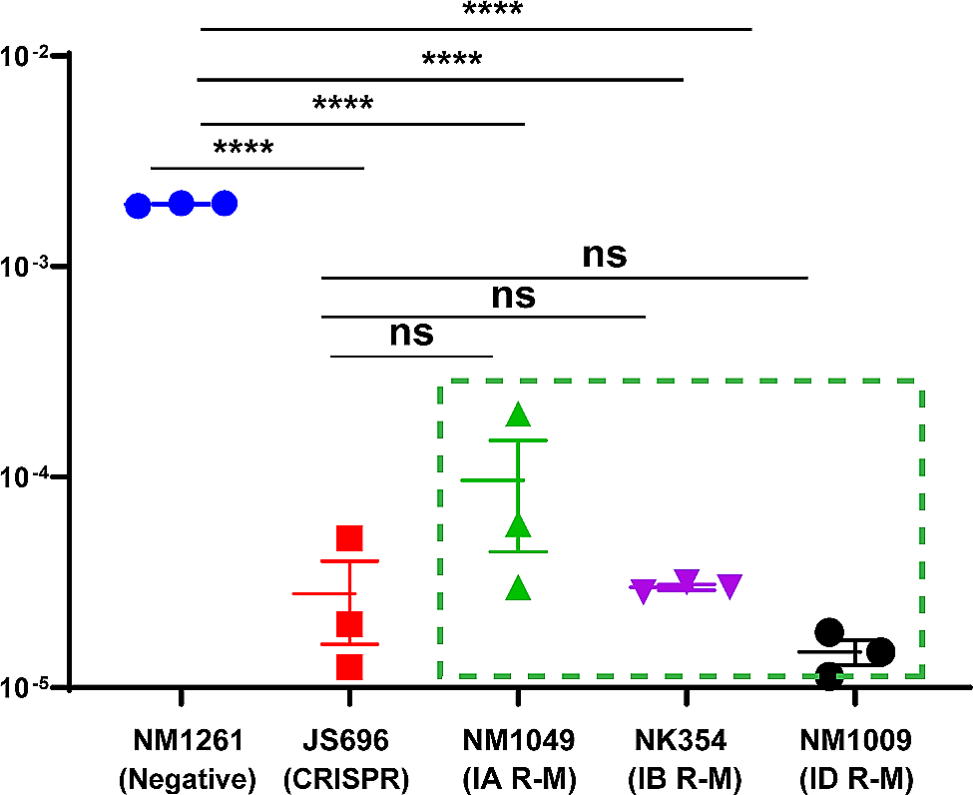
Conjugation frequencies of p187-2 in strains with or without CRISPR-Cas or type I R-M systems. The data represent the mean ± SD for three independent biological replicates. ****p < 0.0001 indicates significant differences between strains harbouring CRISPR or R-M systems and the control group as determined using one-way ANOVA with Dunnett correction. “ns” indicates no significant differences between strains harbouring CRISPR and strains harbouring R-M

The above analyzes suggest that CRISPR-Cas and R-M systems may exist and function independently in some *K. pneumoniae* strains. Although both are recognized as robust defense barriers to incoming DNA, their molecular mechanisms areare quite different, and the functional diversity between them remain unclear in *K. pneumoniae.* Hence, we constructed an isolate harbouring the KP8-CRISPR system (*K. pneumoniae*, CP025636.1, abundant matched spacers) and strains harbouring type IA/B/D R-M systems as recipients to evaluate the discrepancy between these two immune systems in terms of anti-plasmid function. The conjugation results showed that both the CRISPR and R-M systems significantly restricted the invasion of the *bla*_KPC_ plasmid, and both conferred an approximately 2-log reduction in the acquisition of the *bla*_KPC_ plasmid with no significant difference (Fig. 4).

### CRISPR-Cas and restriction-modification systems are compatible and increase plasmid restriction

**Fig. 5:**
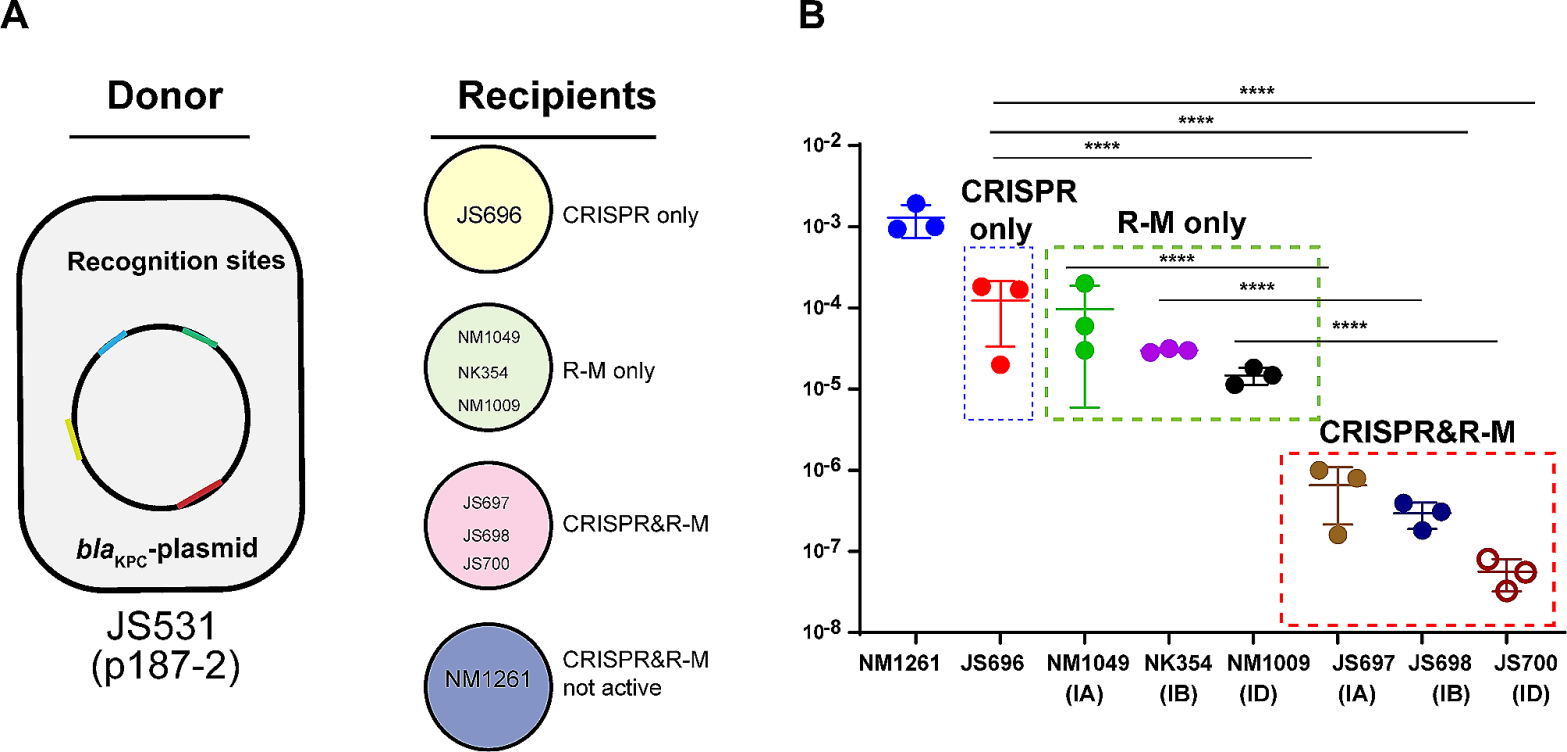
CRISPR-Cas and R-M provide additive defense against pl87-2 plasmid. **(A)** Schematic representation of donor and recipient strains used to assess the individual and collective contributions of R-M and CRISPR-Cas to genome defense. **(B)** Conjugation frequencies of p187-2 in different derivatives strains. Results of these experiments show that the combined effects of CRISPR-Cas and R-M outweigh the effect of either system alone. Data represent results of a minimum of three independent conjugations for all experiments shown. The open circle indicates that no valid conjugant was obtained (JS700, CRISPR&ID R-M). ****p < 0.0001 indicate significant differences between strains and corresponding CRISPR & R-M group as determined using one-way ANOVA with Dunnett correction. E.g., the type IA R-M strains were compared with type IA R-M & CRISPR strains; the CRISPR strains were compared with type IA R-M & CRISPR, type IB R-M & CRISPR, and type ID R-M & CRISPR strains

Next, we sought to determine whether CRISPR-Cas and R-M confer additive genome defense in *K. pneumoniae.* We constructed recipients harbouring I-E CRISPR-Cas (extracted from *K. pneumoniae* KP8) and IA/B/D R-M systems and applied conjugation assays to determine the relative defense contributions. The conjugative experimental design is shown in Fig. 5A. When neither defense system is active, the average conjugation frequency (expressed as transconjugants/donors) is 1.28 × 10^− 3^. We used this value as a reference for comparisons. When CRISPR-Cas defense was compromised, but R-M defense was active, the average conjugation frequency was 2.23 × 10^− 5^, a 57-fold decrease in plasmid transfer. When R-M defense is not active, but CRISPR-Cas defense is active, the average conjugation frequency is 5.13 × 10^− 5^, a 25-fold decrease in plasmid transfer. When both CRISPR-Cas defense and R-M defense were active, the average conjugation frequency was 3.07 × 10^− 7^, a 4167-fold decrease in plasmid transfer (Fig. 5B). Notably, when CRISPR-Cas defense was combined with ID R-M immunity, we did not obtain transconjugants harbouring *bla*_KPC_ plasmid. Overall, we conclude that R-M and CRISPR-Cas, both individually and collectively, have significant effects on conjugative *bla*_KPC_ plasmid transfer.

## Discussion

A correlation between the lack of type I-E CRISPR-Cas or type I R-M systems and multidrug resistance in *K. pneumoniae* has been previously established using genome analysis [3–5]. We found that CRISPR or R-M systems could not completely influence the invasion of some antibiotic resistance genes into *K. pneumoniae.* On their own, they are imperfect barriers to invasion by foreign DNA [3–5]. While some resistance genes can successfully evade restriction conferred by CRISPR or R-M systems alone, these defense systems are rarely present alone in a cell. *K. pneumoniae* has developed multiple defense systems against foreign DNA; however, little is known about whether and how they interact with each other. Here, we studied the connection between these defense systems in *K. pneumoniae* and assessed the coupling anti-plasmid immune function.

We investigated the prevalence of type I R-M and CRISPR in *K. pneumoniae* and found a highly significant inverse correlation between the presence of both functional systems and the acquired *bla*_KPC_ genes in *K. pneumoniae.* Notably, one bacterium always harboured multiple anti-plasmid strategies, and CRISPR-Cas systems usually cohabit with more than one I R-M system in the same host. Although some types of CRISPR-Cas systems (I-E*) and R-M systems (Im6A/IC) may be immunocompromised against *bla*_KPC_ harbouring plasmids, the co-existence of other functional systems (I-E CRISPR and IA/B/D R-M) would compensate for this low-active defense and result in *bla*_KPC_ elimination. Such co-distribution raises the possibility of interactions between R-M and CRISPR-Cas systems. Spacers (CRISPR-Cas) and recognition sites (type I R-M) are key elements of these two system-mediated immunities [3–5]. Hence, both matched proto-spacers and recognition sites harboured by the prominent *bla*_KPC_-IncF plasmids were collected and analyzed. The results indicated that more than one matched sequence from both systems were present in all plasmids. Overall, these findings showed that CRISPR and R-M systems have the potential to achieve *bla*_KPC_ elimination.

Conjugation and transformation assays were used to comprehensively assess the ability of CRISPR and R-M to impede the transmission of *bla*_KPC_ harbouring plasmids. The results demonstrated that CRISPR and R-M harboured by *K. pneumoniae* resulted in effective immunity to *bla*_KPC_ harbouring plasmids containing matched sequences. Each system can successfully cleave DNA at its respective targeted sites with no significant functional differences. Although type I-E CRISPR-Cas and type I R-M had a significant impact on conjugation frequency and transformation rates, they were not perfect barriers to plasmid transfer. Previous studies have demonstrated that CG15, CG147, and other MDR groups can acquire matched resistance plasmids, such as *bla*_CTX−M−15_, successfully evading CRISPR restriction [21, 22]. In R-M systems, defense failures are usually attributed to the pre-methylation of invading resistance genes or the presence of anti-restriction proteins [23, 24]. However, these “*bla*_KPC_-escapers” are rare, as *bla*_KPC_ harbouring plasmids are usually recognized and eliminated by both defense systems in *K. pneumoniae* concurrently, instead of being interfered with by CRISPR or RM alone.

Although two of the most well-studied defense systems often cohabit in the same strain, only a few studies have explored the possibility of interactions between them. It was previously observed that the expression of the type II R-M system in *Streptococcus thermophilus* was compatible with the presence of endogenous type II CRISPR-Cas and provided additive protection against phage infection [24]. Another study using the same experimental setup showed that these systems are not only compatible but also synergistic [25]. Here, we showed that a type I-E CRISPR-Cas immune system is compatible with a type I R-M system in *K. pneumoniae* to increase the inhibition of *bla*_KPC_ plasmid invasion. When both immune systems co-exist in one isolate, the possibility of pre-methylation of the R-M system would not limit the main activities of the CRISPR-Cas system, and R-M defense can still impede plasmid transfer in CRISPR-Cas low-activity cells. Moreover, we also observed that the combined action of both functional systems significantly reduced the likelihood of the emergence of *bla*_KPC_ harbouring plasmids capable of simultaneously evading R-M and CRISPR-Cas systems. Our observation that CRISPR-Cas and R-M defenses individually contribute significantly to anti-plasmid genome defense is consistent with a previous report that the two modes of defense (type II R-M and type II CRISPR-Cas9 systems) work additively against plasmid invasion in *E. faecalis* [26].

However, the molecular mechanism by which the synergy of both systems is achieved remains unexplored. Recently, one study determined the molecular mechanism of the cooperation between type II R-M and type II CRISPR-Cas in *S. aureus* [12]: cleavage of viral DNA by R-M generates double-strand breaks, producing substrates for the acquisition of spacers in CRISPR-Cas systems. This study found that the restriction of endonucleases provides short-term defense, which is rapidly overcome through the methylation of the phage genome. However, restriction results in the acquisition of spacer sequences from the cleavage site, which mediates a robust type II-A CRISPR-Cas immune response against methylated phage. This mechanism is reminiscent of eukaryotic immunity, in which the innate response offers the first temporary line of defense and activates a second and more robust adaptive response. In our study, when both I-E CRISPR-Cas and Type I R-M systems were present in the strain, few *bla*_KPC_-plasmid-escapers were observed, and a long-term anti-*bla*_KPC_-plasmid effect was achieved. In addition to type II, the type I-E system of *Escherichia coli* can use dsDNA ends as preferred substrates for new spacers [27]. These similarities with the spacer-acquisition mechanism of type II CRISPR systems suggest that restriction from R-M would also enhance type I-E CRISPR-Cas responses by incorporating new spacers from restricted DNA. Hence, the anti-plasmid cooperation we observed may also be explained by a similar mechanism, and the presence of a type I R-M system may be a determinant in the acquisition of a new spacer of the type I-E CRISPR system.

Overall, our study demonstrated that the interaction of type I R-M and type I-E CRISPR-Cas systems significantly influences the acquisition of *bla*_KPC_ resistance genes in *Klebsiella pneumoniae.* The limitation of our study is that we only demonstrated the additive effect of CRISPR-Cas and R-M systems against plasmid invasion in *K. pneumoniae* without exploring the related molecular mechanism. Although we had hypothesized the potential molecular mechanism of the synergy of both systems according to previous studies on type II R-M and type II CRISPR-Cas, we would further confirm this mechanism in the future. Moreover, all these findings could not quantitatively determine synergy between CRISPR-Cas and R-M systems, which we would further explored in the future. Antibiotics are used extensively in hospital settings, so we must also consider the impact of CRISPR and R-M systems on the spread of drug-resistant plasmids in an antibiotic environment. In the future, we will need to simulate this environment in vitro.

### Electronic supplementary material

Below is the link to the electronic supplementary material.

Supplementary Material 1

Supplementary Material 2

## Data Availability

All the data and materials were available. The datasets generated and/or analyzed during the current study are available in the NCBI ( https://www.ncbi.nlm.nih.gov/) Database.

## References

[1] Chen L, Mathema B, Chavda KD, DeLeo FR, Bonomo RA, Kreiswirth BN (2014). Carbapenemase-producing Klebsiella pneumoniae: molecular and genetic decoding. TRENDS MICROBIOL.

[2] Fu P, Tang Y, Li G, Yu L, Wang Y, Jiang X (2019). Pandemic spread of bla((KPC-2)) among Klebsiella pneumoniae ST11 in China is associated with horizontal transfer mediated by IncFII-like plasmids. INT J ANTIMICROB AG.

[3] Tang Y, Fu P, Zhou Y, Xie Y, Jin J, Wang B, Yu L, Huang Y, Li G, Li M (2020). Absence of the type I-E CRISPR-Cas system in Klebsiella pneumoniae clonal complex 258 is associated with dissemination of IncF epidemic resistance plasmids in this clonal complex. J ANTIMICROB CHEMOTH.

[4] Zhou Y, Tang Y, Fu P, Tian D, Yu L, Huang Y, Li G, Li M, Wang Y, Yang Z (2020). The type I-E CRISPR-Cas system influences the acquisition of Bla KPC-IncF plasmid in Klebsiella pneumonia. EMERG MICROBES INFEC.

[5] Zhou Y, Tian D, Tang Y, Yu L, Huang Y, Li G, Li M, Wang Y, Yang Z, Poirel L (2020). High-risk KPC-producing Klebsiella pneumoniae lack type I R-M systems. INT J ANTIMICROB AG.

[6] Shen J, Lv L, Wang X, Xiu Z, Chen G (2017). Comparative analysis of CRISPR-Cas systems in Klebsiella genomes. J BASIC MICROB.

[7] Sampson TR, Saroj SD, Llewellyn AC, Tzeng YL, Weiss DS (2013). A CRISPR/Cas system mediates bacterial innate immune evasion and virulence. Nature.

[8] Makarova KS, Wolf YI, Iranzo J, Shmakov SA, Alkhnbashi OS, Brouns S, Charpentier E, Cheng D, Haft DH, Horvath P (2020). Evolutionary classification of CRISPR-Cas systems: a burst of class 2 and derived variants. NAT REV MICROBIOL.

[9] Palacios AD, Palmer KL, Duerkop BA (2021). CRISPR-based antimicrobials to obstruct antibiotic-resistant and pathogenic bacteria. PLOS PATHOG.

[10] Murray NE (2000). Type I restriction systems: sophisticated molecular machines (a legacy of Bertani and Weigle). MICROBIOL MOL BIOL R.

[11] Loenen WAM, Dryden DTF, Raleigh EA, Wilson GG (2013). Type I restriction enzymes and their relatives. NUCLEIC ACIDS RES.

[12] Maguin P, Varble A, Modell JW, Marraffini LA (2022). Cleavage of viral DNA by restriction endonucleases stimulates the type II CRISPR-Cas immune response. MOL CELL.

[13] Cury J, Bernheim A (2022). CRISPR-Cas and restriction–modification team up to achieve long-term immunity. Trends Microbiol (Regular ed).

[14] Grissa I, Vergnaud G, Pourcel C (2007). The CRISPRdb database and tools to display CRISPRs and to generate dictionaries of spacers and repeats. BMC Bioinformatics.

[15] Roberts RJ, Vincze T, Posfai J, Macelis D (2015). REBASE–a database for DNA restriction and modification: enzymes, genes and genomes. NUCLEIC ACIDS RES.

[16] Serfiotis-Mitsa D, Herbert AP, Roberts GA, Soares DC, White JH, Blakely GW, Uhrín D, Dryden DTF (2010). The structure of the KlcA and ArdB proteins reveals a novel Fold and antirestriction activity against type I DNA restriction systems in vivo but not in vitro. NUCLEIC ACIDS RES.

[17] Tang Y, Zhou Y, Meng C, Huang Y, Jiang X (2020). Co-occurrence of a novel VIM-1 and FosA3-encoding multidrug-resistant plasmid and a KPC-2-encoding pKP048-like plasmid in a clinical isolate of Klebsiella pneumoniae sequence type 11. Infect Genet Evol.

[18] Bi D, Zheng J, Li J, Sheng Z, Zhu X, Ou H, Li Q, Wei Q. In Silico Typing and comparative genomic analysis of IncFIIK plasmids and insights into the evolution of Replicons, plasmid backbones, and Resistance determinant profiles. ANTIMICROB AGENTS CH 2018, 62(10). 10.1128/AAC.00764-18 PMC6153814 30012771

[19] Titheradge AJ, King J, Ryu J, Murray NE (2001). Families of restriction enzymes: an analysis prompted by molecular and genetic data for type ID restriction and modification systems. NUCLEIC ACIDS RES.

[20] Roberts GA, Chen K, Cooper LP, White JH, Blakely GW, Dryden DTF (2012). Removal of a frameshift between the hsdM and hsdS genes of the EcoKI Type IA DNA restriction and modification system produces a new type of system and links the different families of type I systems. NUCLEIC ACIDS RES.

[21] Rodrigues C, Desai S, Passet V, Gajjar D, Brisse S. Genomic evolution of the globally disseminated multidrug-resistant Klebsiella pneumoniae clonal group 147. MICROB GENOMICS 2022, 8(1). 10.1099/mgen.0.000737 PMC8914359 35019836

[22] David S, Reuter S, Harris SR, Glasner C, Feltwell T, Argimon S, Abudahab K, Goater R, Giani T, Errico G (2019). Epidemic of carbapenem-resistant Klebsiella pneumoniae in Europe is driven by nosocomial spread. NAT MICROBIOL.

[23] Goldberg GW, Marraffini LA (2015). Resistance and tolerance to foreign elements by prokaryotic immune systems - curating the genome. NAT REV IMMUNOL.

[24] Dupuis M, Villion M, Magadán AH, Moineau S. CRISPR-Cas and restriction–modification systems are compatible and increase phage resistance. NAT COMMUN 2013, 4(1). 10.1038/ncomms3087 23820428

[25] Hynes AP, Villion M, Moineau S (2014). Adaptation in bacterial CRISPR-Cas immunity can be driven by defective phages. NAT COMMUN.

[26] Price VJ, Huo W, Sharifi A, Palmer KL. CRISPR-Cas and Restriction-Modification Act additively against Conjugative Antibiotic Resistance Plasmid Transfer in Enterococcus faecalis. MSPHERE 2016, 1(3). 10.1128/mSphere.00064-16 PMC4894674 27303749

[27] Levy A, Goren MG, Yosef I, Auster O, Manor M, Amitai G, Edgar R, Qimron U, Sorek R (2015). CRISPR adaptation biases explain preference for acquisition of foreign DNA. Nature.

