# Supplementary material for: CRISPR-Cas3 and type I restriction-modification team up against blaKPC-IncF plasmid transfer in Klebsiella pneumoniae
Source: BMC Microbiol. 2024 Jul 3;24:240. doi: 10.1186/s12866-024-03381-7 (PMC11223367; doi:10.1186/s12866-024-03381-7)
Supplement: Supplementary file 2 — Supplementary Material 2 [file 12866_2024_3381_MOESM2_ESM.docx]

**Supplemental material legends**

**Fig S1 Mechanisms of action of CRISPR/Cas immunity**[1]**.**

**Fig S1. Distribution of CRISPR-Cas systems, R-M systems, and *bla*_KPC_ in 459 Chinese clinical isolates**

**Table S1. Oligonucleotides for cloning or PCR**

**Dataset**

**Dataset 1. 932 completely sequenced global *K. pneumoniae* strains used in this study.**

**Dataset 2. 271 *bla*_KPC_ harboring *K. pneumoniae* strains**

**Dataset 3. 249 CRISPR-Cas harboring *K. pneumoniae* strains**

**Dataset 4. 360 Type I R-M harboring *K. pneumoniae* strains**

**Dataset 5 *bla*_KPC_- positive plasmids in 932 *K. pneumoniae***

**Dataset 6 *bla*_KPC_-IncF plasmids in 932 *K. pneumoniae***

**Dataset 7 459 Chinese clinical isolates used in this study**


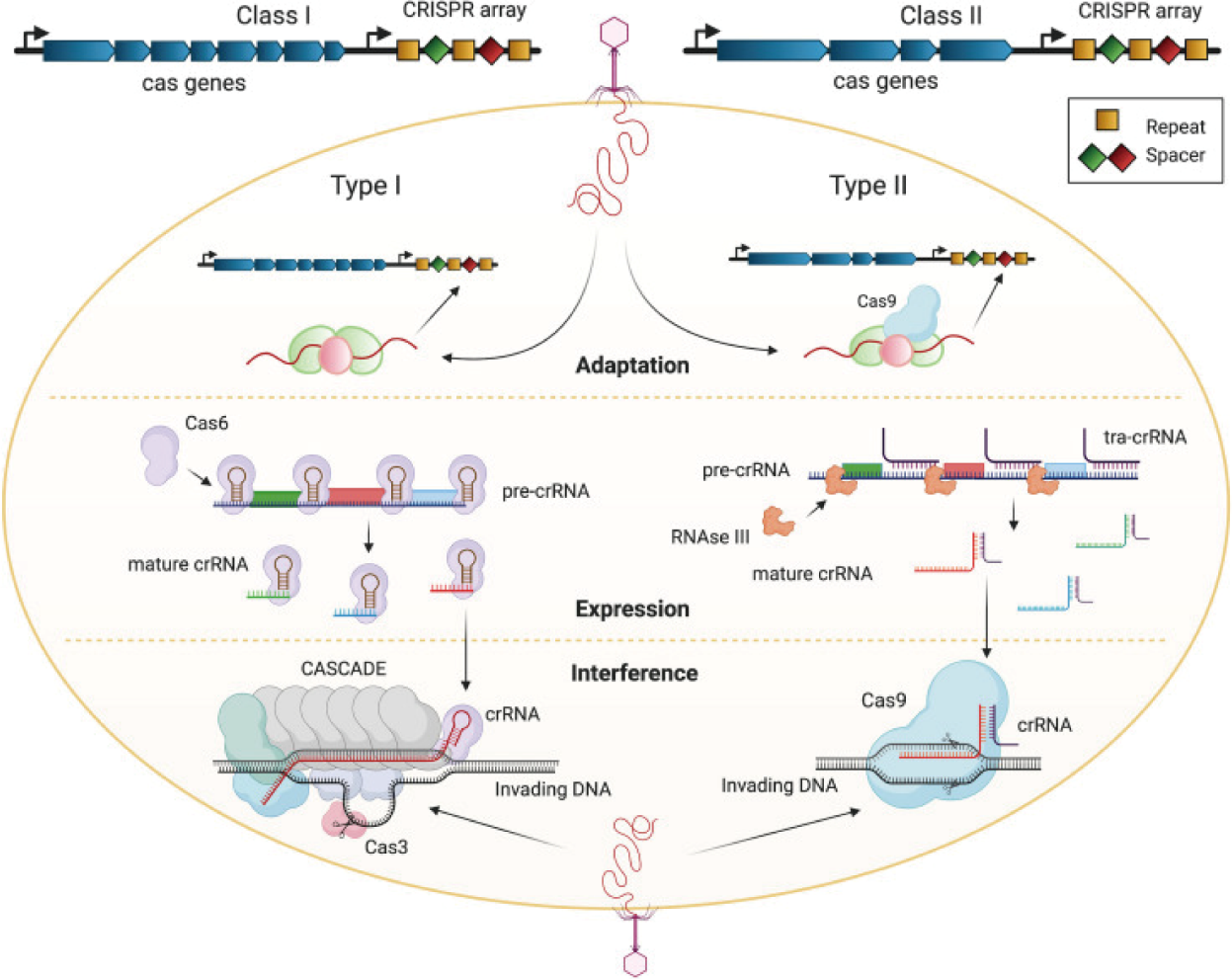


**Figure S1 Mechanisms of action of CRISPR/Cas immunity**[1]**.**

In Class I CRISPR/Cas systems, such as the Type I system, a multi-subunit complex called "Cascade" is used as the effector machinery. In the adaptation stage, the Cas1-Cas2 complex captures a sequence from the invading DNA and adds it to the CRISPR array as a new spacer. In the "expression" stage, the CRISPR array is transcribed into pre-crRNAs, which are then processed into mature interfering crRNAs. During the interference stage, the mature crRNAs direct the Cas proteins to their DNA target. When the crRNA binds to the corresponding DNA target, the Cas protein creates a double-stranded DNA break in the target.
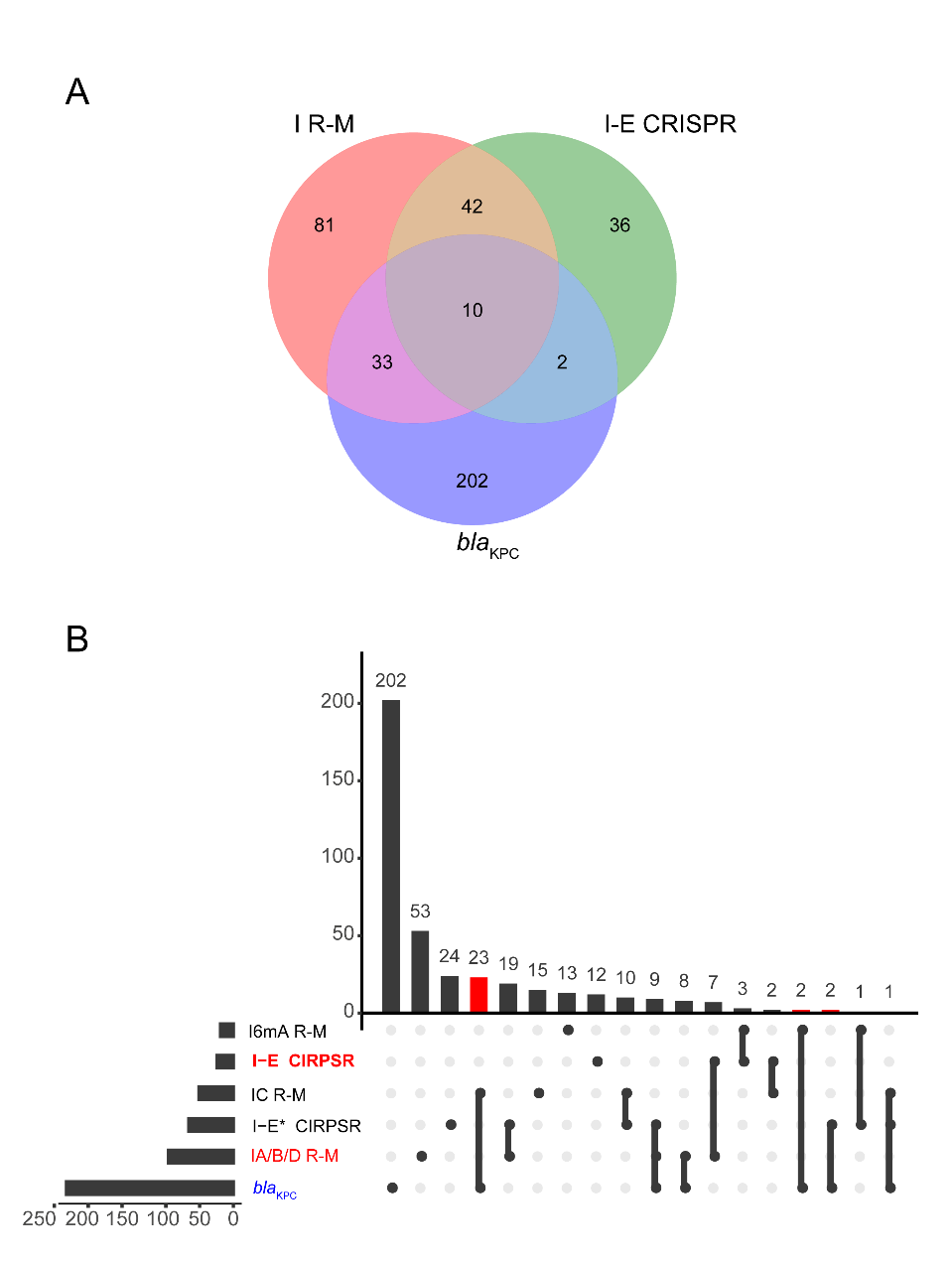


**Figure S2. Distribution of** **CRISPR-Cas systems, R-M systems, and *bla*_KPC_ in 459 Chinese clinical isolates. A. The intersection of different bacteria harboring CRISPR-Cas systems, R-M systems, and *bla*_KPC_ through Venn diagram. B. The detailed information of co-distribution of CRISPR-Cas systems, R-M systems, and *bla*_KPC_ in 459 Chinese clinical isolates**

**Table S1. Oligonucleotides for cloning or PCR**

| Name | Sequence (5'-3')^a^ |
| --- | --- |
| **For CRISPR-Cas screening** |  |
| cysH-iap-F | CGGTTCTTCGGGCTTAAACG |
| cysH-iap-R | CTGCTGCAATGACGCCAG |
| ABC-gly-F | TGTTCGCCGCTGAGTTTATG |
| ABC-gly-R | TACCACGCCAGTTACTACGC |
| cas1-F | CTTTTGGCACGACGGAATCA |
| cas1-R | TGGCGCTGGATGATGATTTG |
| cas3-F | GTCCCGACTAAAATGCGTCC |
| cas3-R | CGTTGATGGCGGTGATGAAT |
| **For *bla*_KPC_ Screening** |  |
| *bla*_KPC_-F | TCGCTAAACTCGAACAGG |
| *bla*_KPC_-R | TTACTGCCCGTTGACGCCCAATCC |
| **For R-M systems screening** |  |
| RM clade1-F | TTGTGGTCAGACAGGCGTTC |
| RM clade1-R | GTTGCAGGAACAGATCCGCC |
| RM clade2-F | TGCTGGTACTTGGATTCCACC |
| RM clade2-R | AGAGCGGCAGTCACAGAAG |
| RM clade3-F | GCGGCGTCTCCTATCAAAAC |
| RM clade3-R | GTGGTCGAGGTTGCCTTCAA |
| RM clade4-F (plasmid-borne) | TCAAGCATACGGACTTTGGC |
| RM clade4-R (plasmid-borne) | GTTTCCGGCTCCAACTCCTG |
| RM clade5-F | CTGAGCCTCGTGTTCCTCAA |
| RM clade5-R | GTTCGAGCATTTCGGTCAGC |
| RM clade6-F (plasmid-borne) | CACCAAACTGAAAGACGCCG |
| RM clade6-R (plasmid-borne) | GCCCGGAGACAATTTTACGC |
| RM clade7-F (plasmid-borne) | CTGATTTCTAACTATGCGGC |
| RM clade7-R (plasmid-borne) | CATGTTCATACGCGCCAGG |
| RM clade8-F | AAGATACGCTGGAGCATCCG |
| RM clade8-R | TGGTTTTATCGATCTCCGCC |
| RM clade9-F | GATTGACGGTTCGGTCTCCA |
| RM clade9-R | GGCATCTTAGCCGGATTGGT |
| RM clade10-F | CCGAGCATGAATTCGCCAAG |
| RM clade10-R | ACGACGTAGTTTTCGTGGCT |
| RM clade11-F | TGATGACCGTCTACGCGATG |
| RM clade11-R | ATTCGATCAGCCACAGGCAA |
| RM clade12-F | CTTGCTTCCTGGGATGAGCA |
| RM clade12-R | TAGCTGGTTACGAAGCTCGC |
| RM clade13-F | ACAACCATCCCTTGGCGAAT |
| RM clade13-R | ACCAGTTGGTAAGCGCAAGA |
| RM clade14-F | AGGTCCGCAATACCAGCAAA |
| RM clade14-R | CGGCGGGTTGGCAAAAATTA |
| **For constructing pACYC-KP8CRISPR** |  |
| pACYC-F | ATATCGCTAGAGCGGCAGGTAGCGATATTT  GGCGGAGATTTCCTGGAAGA |
| pACYC-R | AAATATCGCTACCTGCCGCTCTAGCGATAT GAATTGATTGGCTCCAATTC |
| KP8-CRISPR1-F | ATATCGCTAGAGCGGCAGG |
| KP8-CRISPR1-R | GAGGTACTTCGCCAGGCTTA |
| KP8-CRISPR2-F | GCTGTCGCAGAGAAAGCCTC |
| KP8-CRISPR2-R | GCGCGGTTGTCTCCTCTATT |

^a^ underlines sequences were the overlaps used for plasmid construction

**References:**

1. Palacios AD, Palmer KL, Duerkop BA: **CRISPR-based antimicrobials to obstruct antibiotic-resistant and pathogenic bacteria**. *PLOS PATHOG* 2021, **17**(7):e1009672.
